# Unveiling roles of non‐coding RNAs in cancer through advanced technologies

**DOI:** 10.1002/qub2.70005

**Published:** 2025-04-20

**Authors:** Runhao Wang, Leng Han

**Affiliations:** ^1^ Brown Center for Immunotherapy Indiana University School of Medicine Indianapolis Indiana USA; ^2^ Department of Biostatistics and Health Data Science Indiana University School of Medicine Indianapolis Indiana USA; ^3^ Center for Computational Biology and Bioinformatics Indiana University School of Medicine Indianapolis Indiana USA

**Keywords:** cancer, CRISPR screens, ncRNAs, patho‐DBiT, snoKARR‐seq, spatial transcriptomics

## Abstract

Non‐coding RNAs (ncRNAs) have emerged as key regulators in tumorigenesis. In this perspective, we briefly review the significance of ncRNA in cancer biology and highlight recent technological advancements in characterization of ncRNA in cancer research. Specifically, we discuss how these advanced approaches, such as Patho‐DBiT, CRISPR screens, and snoKARR‐seq, hold the potential to revolutionize ncRNA research by offering comprehensive insights into their spatial expression patterns and functional roles.

## 1 INTRODUCTION

Non‐coding RNAs (ncRNAs) are RNA transcripts that do not encode for a protein. Extensive research has explored the functional roles of various ncRNAs, such as long non‐coding RNAs (lncRNAs), microRNAs (miRNAs), transfer RNAs (tRNAs), small nucleolar RNAs (snoRNAs), and small nuclear RNAs (snRNAs), in human cancers [1, 2, 3]. These ncRNAs play pivotal roles in regulating gene expression, modulating cellular signalling pathways, and influencing critical processes such as proliferation, apoptosis, metastasis, and immune evasion [1, 4]. Their profound impact in cancer biology underscores their potential as diagnostic biomarkers and therapeutic targets. However, the complex mechanisms and spatial dynamics of ncRNAs remain challenging to dissect, necessitating innovative technologies. This perspective briefly reviews the roles of ncRNAs in cancer and highlights technological advancements that enable their functional characterization.

## 2 FUNCTIONAL SIGNIFICANCE OF ncRNAs IN CANCER

ncRNAs are emerging as pivotal regulators in the intricate landscape of gene expression, playing essential roles in cancer research. These ncRNAs influence key cellular processes through diverse regulatory mechanisms, which are briefly summarized in this section with a focus in cancer research. With their functional versatility and broad impact on gene regulation, ncRNAs hold significant promise as biomarkers and/or therapeutic targets in oncology.

### 2.1 lncRNA

lncRNAs (>200 nucleotides) are critical regulators of various biological processes, including tumorigenesis, proliferation, metabolism, and immune responses. In tumorigenesis, lncRNAs modulate oncogenic and tumour suppressor pathways, influencing key processes such as invasion, metastasis and angiogenesis [5]. They also play a central role in metabolic reprogramming by regulating genes involved in glycolysis, lipid metabolism, and mitochondrial function, thereby supporting the metabolic demands of cancer cells [6]. Additionally, lncRNAs control cell cycle progression and apoptosis, acting as either promoters or inhibitors of cell growth, depending on the cellular context [7]. In immune responses, lncRNAs regulate immune cell differentiation, cytokine production, and interactions within the tumour microenvironment (TME), shaping the immune landscape of tumours [8]. lncRNAs often exhibit tissue‐ and cell‐specific expression, and their dysregulation, arising from mutations, deletions, or altered biogenesis, is closely related to cancer progression [9]. For example, lncRNA LINK‐A promotes immune evasion and metastasis in triple‐negative breast cancer by disrupting antigen presentation and tumour suppressor pathways through G‐protein‐coupled receptor signalling, leading to peptide‐loading complex, Rb and p53 degradation, while its inhibition sensitizes tumours to immune checkpoint blockade therapy [10].

### 2.2 miRNA

MicroRNAs (miRNAs) are small ncRNAs (18–25 nucleotides) that post‐transcriptionally regulate gene expression by targeting messenger RNAs (mRNAs) for degradation or translational repression [11]. As oncogenes, miRNAs drive cancer progression by promoting cell proliferation, survival and metastasis through the suppression of tumour suppressor genes [12]. Conversely, they can also act as tumour suppressors by inhibiting oncogenic pathways, thereby reducing tumour growth, and preventing metastatic spread [12]. Dysregulation of miRNAs, whether through mutations, deletions, amplifications or defective biogenesis, significantly contributes to tumour initiation and advancement [13, 14, 15, 16]. Furthermore, transcriptional regulation by oncogenic or tumour‐suppressive factors, such as c‐Myc or p53, integrates miRNAs into the intricate network of cancer biology [17].

### 2.3 tRNA

tRNAs (70–87 nucleotides) not only support protein translation but also contribute to cancer by overcoming translational bottlenecks and promoting oncogenic protein synthesis [18, 19, 20]. Upregulation of tRNAs in cancer cells enhance the synthesis of oncogenic proteins by resolving translational bottlenecks, facilitating rapid tumour growth and proliferation [21]. Alterations in tRNA expression are further coupled with dysregulation of translational enzymes, such as tRNA modification enzymes and aminoacyl‐tRNA synthetases, which amplify translational efficiency and tumour growth [21, 22]. In addition to their functional roles, tRNAs exhibit tissue‐specific expression patterns and have strong associations with clinical outcomes, making them promising biomarkers for cancer diagnosis, prognosis and monitoring disease progression [23]. Dysregulated tRNAs also offer therapeutic potential that targeting abnormal tRNA expression or translational processes may reduce oncogene abundance [24], improve drug sensitivity [25] and even generate neoantigens for immunotherapy [26].

### 2.4 snoRNA

snoRNAs (60–300 nucleotides) are traditionally known for guiding post‐transcriptional RNA modifications, such as 2′‐*O*‐methylation and pseudouridylation, essential for ribosomal RNA and snRNA stability and function [27]. Beyond these canonical functions, snoRNAs actively contribute to cancer by modulating oncogenic signalling pathways, thereby promoting tumour proliferation, survival, and metastasis through interactions with key molecular players [28]. They also influence gene translation by modifying rRNAs and interacting with mRNAs, enhancing the production of proteins critical for tumour growth and progression [28]. Additionally, certain snoRNAs regulate alternative splicing, affecting the production of oncogenic or tumour‐suppressive isoforms and adding complexity to cancer transcriptomes [28]. The dysregulation of snoRNAs has been associated with clinical outcomes [29, 30, 31, 32, 33, 34], positioning them as promising biomarkers for cancer diagnosis, prognosis and therapeutic targets.

### 2.5 snRNA

snRNAs, approximately 150 nucleotides in length, are essential components of spliceosomes and play a critical role in RNA splicing [35]. Emerging evidence highlights that dysregulation of snRNAs in cancer leads to altered splicing patterns, resulting in the production of oncogenic transcripts while reducing the expression of tumour suppressors, thereby driving tumorigenesis [36]. Mutations or aberrant expression of snRNAs can disrupt normal alternative splicing, affecting key cancer signalling pathways such as cell cycle regulation, apoptosis, DNA repair, PI3K/AKT and mitogen‐activated protein kinase, promoting tumour growth and metastasis [37, 38, 39]. Additionally, snRNAs have been associated with clinical outcomes, including cancer prognosis and therapeutic response, making them valuable biomarkers for cancer diagnosis and treatment monitoring [40, 41]. Targeting dysregulated snRNAs or correcting their associated splicing errors offers a promising avenue for anti‐cancer strategies, potentially restoring normal splicing and inhibiting oncogenic pathways [42].

## 3 TECHNOLOGICAL ADVANCEMENTS FOR CHARACTERIZATION OF ncRNAs

**FIGURE 1:**
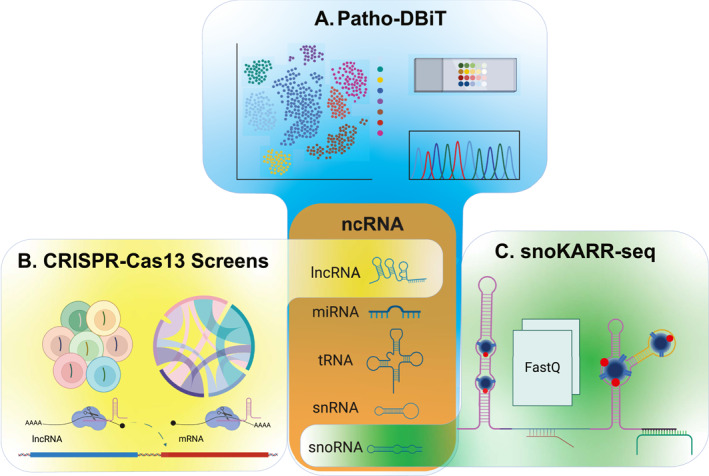
Overview of advanced technologies for ncRNA research in cancer biology. (A) Patho‐DBiT enables high‐sensitivity profiling of diverse RNA species, uncovering patient‐specific vulnerabilities. (B) RNA‐targeting CRISPR‐Cas13 screens systematically perturb lncRNAs with strand and transcript specificity, identifying and comparing essential lncRNAs involved in different human cell lines. (C) snoKARR‐seq employs chemical crosslinking to identify transcriptome‐wide snoRNA‐mRNA interactions, revealing noncanonical functions of snoRNAs in mRNA dynamics relevant to cancer progression. KARR‐seq, kethoxal assisted RNA‐RNA interaction sequencing; lncRNAs, long non‐coding RNAs; mRNA, messenger RNA; ncRNA, noncoding RNA; snoRNAs, small nucleolar RNAs.

The functional analysis of ncRNAs has been constrained by technical limitations, particularly in degraded formalin‐fixed paraffin‐embedded (FFPE) tissues, which are essential for clinical diagnostics [43]. Recent innovations are transforming ncRNA research and enabling unprecedented insights into their roles in cancer (Figure 1).

### 3.1 ncRNA‐spatial transcriptomics (ST)

ST combines spatially resolved molecular profiling with transcriptome‐wide analysis, enabling the precise mapping of RNA expression within intact tissue environments [44, 45, 46, 47]. This approach enhances our understanding of tumour heterogeneity, therapeutic resistance and the role of the TME in disease progression [48, 49, 50]. Recent advancement Patho‐DBiT extends the power of ST by enabling high‐sensitivity profiling of diverse RNA species in FFPE tissues [51]. By combining polyadenylation with spatial barcoding, Patho‐DBiT accurately maps a broad range of ncRNAs. For example, Patho‐DBiT enhances gene body coverage and significantly increases 5' untranslated region read mapping, enabling the detection of a diverse range of ncRNAs [51], including lncRNAs, miRNAs, tRNAs, snRNAs, and snoRNAs (Figure 1A). This technology has been instrumental in reconstructing spatial cellular compositions and uncovering molecular pathways in archival cancer tissues, bridging histopathology and genomics. Patho‐DBiT’s ability to reveal region‐specific ncRNA activities, tumour vulnerabilities, drug‐resistant clones and patient‐specific targets positions it as a transformative tool in precision oncology. By overcoming technical barriers associated with FFPE tissues, it enables high‐resolution exploration of ncRNA biology, providing unprecedented insights into ncRNA‐mediated mechanisms and tumour heterogeneity.

### 3.2 RNA‐targeting CRISPR screens

CRISPR interference (CRISPRi) and CRISPR activation (CRISPRa) are CRISPR‐Cas9‐based technologies designed for gene regulation [52, 53]. CRISPRi employs a catalytically inactive Cas9 (dCas9) fused with a transcriptional repressor to silence gene expression at specific genomic loci [52], while CRISPRa uses dCas9 fused to transcriptional activators to enhance gene expression [53]. These approaches have proven invaluable for identifying functional lncRNAs by modulating their expression levels. Recently, the development of CRISPR‐Cas13, an RNA‐targeting system, has revolutionized lncRNA screening [54] (Figure 1B). Unlike DNA‐based perturbation, CRISPR‐Cas13 directly targets lncRNA transcripts with strand‐ and transcript‐specific precision, minimizing unintended effects on neighbouring genes or genomic elements. By perturbing about 6200 lncRNAs across multiple human cell lines, these screens have identified hundreds of lncRNAs with context‐specific or broad essentiality. For example, they identified cell‐type‐specific essential lncRNAs, *XLOC_013548* (essential in HAP1) and *XLOC_044949* (essential in THP1), which showed variable expression across cell lines [54]. In contrast, broadly essential lncRNAs exhibited more consistent expression and depletion patterns [54]. Importantly, many essential lncRNAs operate independently of nearby protein‐coding genes, emphasizing their unique functional roles.

### 3.3 snoKARR‐seq

Kethoxal assisted RNA–RNA interaction sequencing (Figure 1C) is a cutting‐edge technique that uses N_3_‐kethoxal labelling and multifunctional chemical crosslinkers to covalently capture RNA‐RNA interactions and higher‐order RNA structures directly within cells, independent of local protein binding [55]. This method provides highly sensitive and accurate detection of widespread intermolecular RNA–RNA interactions and RNA folding dynamics. Building upon this foundation, snoKARR‐seq, a recent breakthrough, extends these capabilities to systematically identify RNA targets of snoRNAs across the transcriptome [56]. This chemical crosslinking‐based approach has uncovered thousands of previously unrecognized snoRNA‐mRNA interactions, revealing non‐canonical snoRNA functions beyond their traditional roles in RNA modifications. Notably, snoKARR‐seq has demonstrated that snoRNAs can regulate mRNA dynamics and enhance the secretion of secretory and membrane proteins, processes highly relevant to cancer progression and the TME. For example, *SNORA73*, one of these snoRNAs, targets mRNAs encoding secretory and membrane proteins and interacts with *7SL* RNA, a component of the signal recognition particle (SRP) essential for protein secretion. The mRNA‐*SNORA73*‐*7SL* RNA interactions strengthen the association between *SNORA73*‐targeted mRNAs and SRP, thereby enhancing the secretion of the encoded proteins [56].

## 4 DISCUSSION AND PERSPECTIVE

Recent advancements in ST, RNA‐targeting CRISPR screens and snoRNA‐target profiling have revolutionized ncRNA research in cancer (Figure 1), providing unparalleled insights into their roles in tumour heterogeneity, progression and therapy resistance. Integrating these technologies with multi‐omics approaches and advanced computational tools promises to enhance the understanding of ncRNA‐mediated mechanisms and facilitate the identification of clinically actionable targets.

## AUTHOR CONTRIBUTIONS

**Runhao Wang:** Investigation; visualization; writing—original draft. **Leng Han:** Writing—review and editing; project administration; supervision.

## CONFLICT OF INTEREST STATEMENT

The authors declare no conflicts of interest.

## ETHICS STATEMENT

This article is a perspective article and does not contain any studies with human or animal subjects performed by any of the authors.

## Data Availability

The authors have nothing to report.
